# Dr. Forbes and Homœopathy

**Published:** 1846-09

**Authors:** 


*Dr. Forbes and Homœopathy.*—In our last number we gave the conclusion of Dr. Forbes's explanation of his article on Homœopathy which, as we stated, had elicited a great deal of remark from his professional brethren. We append the whole of his paper not already inserted, and are sure it will be read with interest.

The article in the last number of this Journal [*British and Foreign Medical Review*] entitled 'Homœopathy, Allopathy, &c.,' as was, perhaps, to be expected from its subject and object, has attracted a more than ordinary degree of attention from the profession. The editor has, in consequence, been favored with numerous communications relating to it from his friends in all parts of this country, as well as from the continent of Europe, and from America. The views of the writers are, on the whole, in remarkable accordance with those given in the article; and the opinion is generally and strongly expressed, that some such changes in the manner of studying and treating diseases as are there propounded, are absolutely necessary for the successful progress of rational medicine. So

*Made thus: ℞ Equal parts of sulph. iron and carb. potas, powder separately and mix intimately; make into pills of six grains; of which 2 to be taken thrice daily for 3 days; 4 as often for as many days; and then 6 three times daily till cured.

convinced is the editor (the author of the article in question) of the truth and importance of this subject generally, that he now offers to open the pages of his Journal for its special consideration, if it shall be found that the warm interest at present excited by it, proves to be deep and strong enough to supply materials for its continuous elucidation and advancement. This is doubted. Still it has been thought advisable to evince practically and without delay, the earnestness with which the subject has been taken up and is sought to be promoted by the editor. From among the numerous contributions with which he has been already favored, two have, with the sanction of their authors, been selected for publication in the present number. Whether these shall be followed by others, in subsequent numbers, will, as just stated, depend on the inclination of the profession. It will afford the editor much satisfaction to be able to present to his readers any authentic communications of the same high stamp and admirable tendency as those now submitted to their notice.

Before concluding this prefatory notice, the author of the article on Homœopathy and Allopathy desires to make a few brief observations in reference to one or two points in it, on which he finds he has been somewhat misunderstood. The misconception is admitted by him to be natural on the part of his readers, in consequence of the necessity under which he found himself, for want of both room and time, of leaving all the latter part of the article a mere sketch or outline. Had he completed the whole subject on the same scale as was allowed to the exposition of the character and fallacies of homœopathy, he believes that he would have left no grounds for the friendly criticisms and gentle remonstrances which some of his very estimable and enlightened correspondents have addressed to him. Most assuredly, in this case, he would have run no risk of being set down—as he finds he has been set down by a few of his more careless readers—as favoring homœopathy; as denouncing rational therapeutics on the old system; as sceptical of the powers of all medicines; and as desirous of having diseases generally abandoned to the influences of Nature.

With regard to the charge of favoring homœopathy, it is submitted that it is the first duty of the historian to give an impartial narrative of the events and a fair exposition of doctrines, without prejudice or affection. This, it is believed, was done. And if full credit was given by the author of the article to the statements in the documents submitted to his consideration, and full justice rendered to the characters of the individuals concerned, this was done—simply because it was felt to be just and right so to do. The writer might be mistaken in the estimate he formed of the value of the premises, but having conscientiously formed that estimate, its expression was a matter of course. The obligation of candor, imposed on all inquirers, was the more imperative in the present case, inasmuch as the writer felt that he stood in the position of a declared antagonist. And he knew, moreover, that anything approaching to unfairness could, in the end, only tend to injure its author and the cause he advocated. The writer was, indeed, too profoundly impressed with the reality and value of the ordinary system of medicine (miscalled in the article *allopathy*, to avoid circumlocution) to entertain any fears from conceding to homœopathy everything which it could possibly claim of right. And he cannot help indulging the belief that the arguments adduced against the validity of its doctrines will be found to be more efficacious than many previously urged, simply because more candor has been shown in examining these doctrines, and more justice done to the characters of the men who have originated and professed them.

Probably the statement in an early part of the article, to the effect that, 'homœopathy might be the remote, if not the immediate cause, of more important fundamental changes in the practice of the healing art, than have resulted from any promulgated since the days of Galen himself,' made an impression on some readers who did not attend to the qualifications afterwards given. The 'fundamental changes' alluded to had exclusive reference to the indirect or negative operation of homœopathic practice, in furnishing us with 'the means of instituting a grand natural experiment in therapeutics,' by leaving diseases really to nature, though nominally to medicinal treatment. And it is a profound conviction of the great importance of the knowledge to be thus obtained, which makes the writer now reiterate the statement, as well as another in a subsequent part of the article to the same effect, viz., 'that the doctrine of Hahnemann will have [*thus*] conferred an inestimable benefit on the healing art.' It never was intended to be conveyed as the opinion of the author, that the homœopathic treatment was valuable in itself, or that the system of rational eclectic medicine advocated by him, could derive any direct benefit from homœopathy by the adoption of any part of its so called medicinal therapeutics.

But, the writer thinks, moreover, that the declaration of his opinions, in more than one part of his paper, was sufficiently positive, clear, and forcible, to secure him from the charge of favoring homœopathy in any way, or of over-estimating it at the expense of rational medicine. For instance, having in the course of the investigation come to the conclusion that homœopathy is to be utterly rejected and the old faith still adhered to, he added: 'In doing so, we consider, that though we are embracing a system extremely imperfect, we are, at least, embracing one which, with all its faults, contains a considerable amount of truth, and yet a greater amount of good; and which, above all, is, or may be made, in its exercise, consonant with the principles of science, and is capable of indefinite improvement; while, *in rejecting homœopathy, we consider that we are discarding what is, at once, false and bad—useless to the sufferer and degrading to the physician.*' (p. 250.) And again: 'It may possibly be inferred that we are entirely sceptical of the truth of medicine as a science, and think most meanly of it as a practical art. And yet this is not so. On the contrary, we look upon medicine, regarded in all its parts and all its bearings, as a noble and glorious profession, even in its present most imperfect state; and we believe it destined to become as truly grand and glorious in actual performance, as it now is in its essence, its aims, and its aspirations.' p. 259.)

A more specific charge has been made against the author for admitting, without sufficient coroborative evidence from other quarters, the validity of the results given in Dr. Fleischmann's Report. He anticipated this charge, and endeavored to guard himself, in some degree, against it, by stating that while he allowed Dr. Fleischmann to be a man of honor and respectability, and incapable of attesting a falsehood, he could only admit his statements 'with that liberal subtraction from the favorable side of the equation, which is required in the case of all statements made by the disciples and advocates of new doctrines.' (p. 242.) The 'subtraction' necessary in every individual case, assuming the narrators to be honest, will depend on many circumstances, both personal and relative. The author was and is disposed to make it very ample in the present case—so ample he believes, as would deprive Dr. Fleischmann and homœopathy of all the glory they have derived from this flattering record. Nevertheless, he here repeats the assertion that, 'even after this rectification, enough remains to justify the inference that these tables substantiate the momentous fact, that all our ordinary curable diseases are cured [*i. e.* get well] in a fair proportion, under the homœopathic method of treatment.' (p. 242.) The expression 'fair proportion' is very indefinite, and therefore improper; it was meant to have reference to the results obtained under the *more general or common* modes of treatment pursued by practitioners, and which it was one main object of the article to denounce. It never was stated, nor was it in any degree believed, that the results obtainable and obtained under homœopathic treatment were equally favorable with those obtainable and obtained under an *improved system* of eclectic treatment, which it was admitted, was now practised by many, and the more general extention of which among practitioners it was the great aim of the paper to promote.

The writer of the article, however, may now state that, since the publication of this paper, he has received from one of our most distinguished clinical physicians a corroboration of a part of Dr. Fleischmann's report which has been regarded with much scepticism. This gentleman reports that he has over and over again proved the complete curability of *pneumonia* when left to the powers of nature, in cases under his own immediate observation. These cases occurred in persons who had come into the hospital in so advanced a stage of the disease, and with symptoms so favorable, as not only to justify but to render imperative the propriety of not subjecting them to any formal treatment. And the result, as stated, was perfect recovery.

In dwelling so long, and expressing himself so strongly on the curative powers of nature, the design of the author of the article was to remove all rational doubt of the capacity of the *vis medicatrix* to effect all that was effected under the imaginary influence of homœopathy. And he had, also, the great collateral object in view of doing his best to stay the plagues of polypharmacy, medical heroism, and many other kinds of *hurtful* interference with the progress of disease, which he believed and believes to be widely diffused in practice, and which loudly demand the attention of all who wish well to the best interests of the medical profession and the public. And he still thinks that, with so legitimate a purpose, he made no statements that are not only justifiable but imperiously called for. Nothing, however, could be further from his intention than to advocate the leaving of diseases to nature, as a general rule, or as a rule at all; or to deny the great value and efficacy of remedies, whether drugs or other means, in the treatment of many of them. The whole of his argument and invective was against the *abuse*, not the rational and legitimate use of medicines and medical treatment. So far from wishing all diseases left entirely to nature, the author of the article has the strongest conviction that none ought to be so left; and that there is not a single case, whatever be its nature, and whether curable or not, that will not be benefited, more or less, by the careful superintendence of the scientific physician. For a man thoroughly acquainted with diseases in all their relations there will always be enough to do in regulating, as far as practicable, the manifold circumstances and conditions amid which the sick are placed, even if the individual case should require little or no medicine in the form of drugs. It is a most mean and circumscribed view of therapeutics, to regard it as almost identical with the use of drugs. So far is this from being the case, that it may almost be said to be the perfection of medical treatment to cure diseases without drugs, or with the smallest possible amount of drugs; as it is now allowed to be the perfection of surgery, not to perform operations but to do away with their necessity. And yet what an almost boundless variety of things, exclusively of drugs, call for the notice, and attention, and consideration, and discrimination, and appreciation of the man who is held responsible for the proper management of a severe disease, whether acute or chronic? It would, indeed, be strange if a task like this were either facile or of slight importance; that it could be safely entrusted to any one not deeply versed in all the better parts of medical science; or that its well or ill performance were a matter of indifference to the patient.

